# Diagnostic-Quality Guided Wave Signals Synthesized Using Generative Adversarial Neural Networks

**DOI:** 10.3390/s22103848

**Published:** 2022-05-19

**Authors:** Mateusz Heesch, Michał Dziendzikowski, Krzysztof Mendrok, Ziemowit Dworakowski

**Affiliations:** 1 Department of Robotics and Mechatronics, AGH University of Science and Technology, Al. A. Mickiewicza 30, 30-059 Krakow, Poland; heesch@agh.edu.pl (M.H.); mendrok@agh.edu.pl (K.M.); 2 Airworthiness Division, Air Force Institute of Technology, ul. Ks. Boleslawa 6, 01-496 Warsaw, Poland; michal.dziendzikowski@itwl.pl

**Keywords:** guided waves, structural health monitoring, neural networks

## Abstract

Guided waves are a potent tool in structural health monitoring, with promising machine learning algorithm applications due to the complexity of their signals. However, these algorithms usually require copious amounts of data to be trained. Collecting the correct amount and distribution of data is costly and time-consuming, and sometimes even borderline impossible due to the necessity of introducing damage to vital machinery to collect signals for various damaged scenarios. This data scarcity problem is not unique to guided waves or structural health monitoring, and has been partly addressed in the field of computer vision using generative adversarial neural networks. These networks generate synthetic data samples based on the distribution of the data they were trained on. Though there are multiple researched methods for simulating guided wave signals, the problem is not yet solved. This work presents a generative adversarial network architecture for guided waves generation and showcases its capabilities when working with a series of pitch-catch experiments from the OpenGuidedWaves database. The network correctly generates random signals and can accurately reconstruct signals it has not seen during training. The potential of synthetic data to be used for training other algorithms was confirmed in a simple damage detection scenario, with the classifiers trained exclusively on synthetic data and evaluated on real signals. As a side effect of the signal reconstruction process, the network can also compress the signals by 98.44% while retaining the damage index information they carry.

## 1. Introduction

### 1.1. Guided Waves in Structural Health Monitoring

Structural health monitoring (SHM) is widely researched [1,2,3], as the viability of continuously monitoring an object offers many potential benefits, such as cutting down maintenance costs by ensuring the maintenance schedule is based on the actual condition of the elements and early detection of material deterioration and damage in various structures. One such widely-researched method is based on guided wave (GW) signal analysis [4,5,6,7,8], in which the propagation of transducer-induced vibration along natural boundaries of a structure is investigated for signs of fatigue or damage. This method has numerous strong points, such as being able to monitor large structures with relatively few transducers and its sensitivity to small changes [4]. Unfortunately, analyzing these output signals is a non-trivial task due to the complexity of the output. This naturally motivates research in applying the latest advances in machine learning for processing and interpreting these signals [9,10,11,12,13,14]. However, machine learning generally requires vast amounts of data to be appropriately trained.

### 1.2. Data Scarcity

High-volume data acquisition of GW is often unfeasible, especially when recording various damage states for the damage detection to be trained on. Doing this will generally require introducing various damage states to the measured structures, which are potentially costly, irreplaceable, or otherwise important. This data scarcity problem is not unique to GW and is present across various SHM problems [2]. In a broad sense, this issue is not even unique to SHM; due to the importance of the quality of data supplied to machine learning algorithms, many fields of study struggle with data collection appropriate for the extensive utilization of these algorithms [15]. This common problem has sparked research into circumventing insufficient data by adding synthetic examples, such as data augmentation, which multiplies the available data via a series of transformations that result in different, but still valid examples. For instance, in the case of image recognition tasks, one might randomly blur, rotate, and add noise to the images, as none of these operations change the fundamental contents of the image, but they do result in distinct examples for the training algorithms. Shorten and Khoshgoftaar [15] have written a comprehensive review on this topic, investigating both simple and complex augmentation methods.

Amongst these more complex methods of creating synthetic data samples, generative adversarial networks (GANs) [16] are a promising tool. In their basic form, they are a pair of networks that learn to re-create the distribution of data they were supplied with during training. Since their inception, there has been significant research into their potential uses [17]. Though the original implementations were focused on working with images, the underlying principles have since been applied to various other types of data, such as audio waves [18]. Moreover, their utility as a data-augmentation tool with a positive effect on machine learning models has been proven in many research fields [15], helping with problems such as kidney CT scan segmentation [19], machine fault detection [20], or card fraud detection [21].

### 1.3. Contribution

This work tackles the application of GANs to generate synthetic signals of a quality sufficient for usage in designing and training damage detection algorithms, extending the previous work on GW-GAN [22] (guided wave—generative adversarial network) a generative adversarial neural network model for GW based on the state-of-the-art architecture for image synthesis StyleGAN2 [23,24]. The motivation behind this work is the difficulty of obtaining guided waves data of sufficient quality, quantity, and distribution [2] to adequately explore applications of some of the newer machine learning (ML) advances in GW analysis. This aim was further bolstered by the successful applications of GANs in various other fields for data synthesis and augmentation, most notably audio signals, which are close to guided waves in that both are mechanical waves. This research is intended to provide background for future StyleGAN applications to synthesize particular types of data for GW-based damage detection, including previously unseen sensor locations or generation of artificial damage in various structures. To the best of the authors’ knowledge, style-based GANs have not yet been used for time-series signal synthesis, and while this architecture was designed with guided waves in mind, it should work with other time-domain signals, possibly requiring minor alterations depending on the nature of these signals. For the guided waves proof of concept, the model was trained using the OpenGuidedWaves dataset [5].

## 2. Materials and Methods

### 2.1. Guided Waves

Guided waves are elastic waves that propagate through a structure, “guided” by its physical boundaries, e.g., along a rail or pipe. Their propagation is highly dependent on the medium they are in and on the environmental conditions, including, most notably, temperature. They are highly dispersive waves and can generate nearly infinite modes by longitudinal and transverse waves superposition [25]. They also retain most of their energy when traveling long distances, making them suitable for monitoring large structures. GWs are usually used at ultrasonic frequencies and, due to that, are often referred to as ultrasonic guided waves.

#### 2.1.1. Damage Detection via GW

GW-based structure monitoring requires introducing the wave into the structure and then measuring it at a different position to investigate its propagation [26]. The sensors are most commonly used in two configurations: “pitch-catch” or “pulse-echo”.

In the former configuration, the monitored area is set between the actuator and sensor. If anything happens to that area, the propagation of this wave will be altered, resulting in various changes to characteristics such as signal energy, impedance, or amplitude. Such a configuration allows one to set up a network of transducers on a monitored surface. All of the transducers sequentially act as the “pitcher”, while the remaining ones “catch”, resulting in a dense network of monitored paths.

In the latter method, the signal is generated and then sensed by the same sensor or by a group of sensors located in a close neighborhood. The principle involves sending a signal to the structure and then listening for reflections that the presence of damage might have caused.

#### 2.1.2. Simulating GW Signals

Modeling of Lamb wave propagation is a vast subject on its own. Elastic waves traveling in thin-walled structures are dispersive in nature; furthermore, for every frequency, at least two fundamental modes of Lamb waves with different propagation speeds can be excited, which makes signals acquired by PZT sensor networks particularly challenging [27,28]. Therefore, from the beginning of PZT sensors’ application for structural health monitoring, the development of numerical and analytical tools for a better understanding of elastic wave propagation in thin-walled structures was of the utmost importance [29]. Over the years, numerous approaches to this topic have been proposed and many excellent papers [29] and books [30] providing detailed reviews of the state-of-the-art in numerical wave propagation analysis have been published. While a detailed and comprehensive discussion of different approaches to this problem is beyond the scope of this paper, a short subjective summary of developments in the field is provided in this section.

The common reasoning behind the justification of different methods is related to the particular trade-off between the computational efficiency of a given approach and its accuracy, especially in numerical model development for more realistic structures. Remarkably, significant insight into such a complex phenomenon was achieved with the application of analytical methods to elastodynamic equations for thin-walled waveguides [27]. In particular, the problems of fine-tuning of frequencies optimal for selective excitation of fundamental symmetric $S_{0}$ or antisymmetric $A_{0}$ Lamb wave modes with PZT ceramic sensors [31,32] and the determination of guided waves’ reflection and transmission coefficients from cracks [33] and delaminations [34] were solved by utilization of exact methods. The exact approach is still of interest in the community, as it provides the most in-depth understanding of various phenomena and also does not require a large amount of computational power. Therefore, it is possible to develop dedicated software for the design and optimization of SHM systems based on PZT sensor networks that can run on a regular desktop PC [35]. Some of the latest developments of the approach allow for simulation of guided waves’ interaction with various types of damage, e.g., notches, cracks, or disbonds [36]. Since analytic methods have proven to be very accurate for the simulation of guided wave propagation for pristine structures and simple damage models, combined semi-analytic approaches were also proposed [29], e.g., the combined analytical finite element model approach (CAFA) [35]. In such approaches, exact solutions are usually used to simulate guided waves’ propagation in the part of the structure without damage or other discontinuities. Interaction of elastic waves with damage is then simulated using a detailed numerical approach, e.g., the finite element (FE) method based on the analytically-derived wave field distribution on the boundary of the damaged area.

In parallel, numerical, computationally-demanding approaches were developed, which can simulate complex interactions of guided waves with structure discontinuities in great detail. For complex scenarios, standard implementations of numerical algorithms for solving differential equations became insufficient for guided wave simulation [29]. Different frameworks of numerical solutions to elastodynamic equations were followed; e.g., the finite difference (FD) scheme augmented with physically-motivated heuristic rules of wave interaction at interfaces resulted in a successful implementation called the local interaction simulation approach (LISA) [37,38]. However, the most often used are simulation schemes based on the finite element method and its modifications, tailored to specific issues of guided wave propagation simulation [29,39], e.g., the spectral element method [30,40], boundary element methods [41], wave finite elements (WFE) [42], and the elastodynamic finite integration technique (EFIT) [43,44].

Profound advancements in understanding the mechanisms of guided waves’ propagation and their interaction with damage were achieved due to the application of various simulation approaches, which makes them indispensable in the design and optimization of SHM systems based on PZT networks. Nevertheless, the second ultimate goal of their application, which is the augmentation of databases of signals acquired in real experiments, is still not fully reached, especially in the case of SHM application to realistic structures and damage models. There are examples of purely numerical approaches to study and design methods of signal analysis or classification, and their validation is based on experimental data [45,46]. However, full reconstruction of real signals acquired by PZT sensors to replace true signals for the purpose of data classification model training is a very demanding task, especially in the case of real structures or complex damage scenarios. At the cost of significant computational power requirements, numerical methods allow for complex real case scenario simulation. Recent advances covers in particular:

- Guided waves scattering on impact damage of composite structures [43,47] or delaminations [48];
- Transmission of guided waves across partially-closed cracks [49];
- Wave damage interaction coefficients for lightweight structures [10];
- Damage of reinforced concrete beams [50];
- Looseness of joint structures in cylindrical waveguides [51];
- Matrix cracking in laminated composites [52].

**Figure 1:**
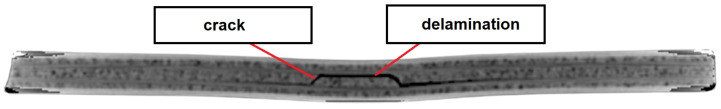
Cross section visualization of impact damage of a composite structure obtained with use of computer tomography [54].

In real case scenarios, the main challenge from a model definition perspective is the proper characterization of the materials as well as the definition of all of the contact conditions, e.g., for structure discontinuities caused by damage or which are naturally present, adhesive bonds of sensors with the substrate material, or when some structure reinforcements are present. Furthermore, additional factors can contribute to signals acquired by PZT sensors, e.g., properties of particular PZT ceramics, the true distribution of the electric field used for sensors excitation, external measurement conditions, or the characteristics of electronic devices used for signal acquisition. This makes the problem of signal reconstruction even more challenging due to the lack of input data to a given particular model; therefore, normalization of numerically-obtained signals is usually required for true data comparison. In spite of the numerous issues mentioned above, proper application of numerical models can provide accurate results, even for very challenging tasks. In [43], the authors presented a very detailed approach to simulation of guided wave scattering on barely visible impact damage (BVID) of composite structures, utilizing CT scans for very accurate 3D damage assessment. BVID forms subsurface transverse cracks of matrix layers and multiple delaminations in the composite (see Figure 1), which makes them particularly demanding for simulation due to the presence of discontinuities where guided wave scattering and different modes excitation may occur. Although the obtained results are qualitatively remarkably accurate, some important components of the simulated wavefield are missing as compared to the real case.

Numerical methods have proven to be a reliable vast source of data for the purpose of development of data-driven approaches to structural health monitoring based on guided wave propagation [11,53]. Recent developments of artificial intelligence algorithms and their successful application in different tasks have opened yet another perspective to address this issue. In this paper, the application of deep generative adversarial networks (GANs) for the reconstruction of full, non-normalized signals is demonstrated. The application of GANs to this problem provides little or no insight into the fundamentals of guided wave propagation; however, GANs can be very efficient in mimicking the full details of signals acquired by PZT transducers, taking into account all of the factors of the experimental setup, which allows their application for classification model training and validation, fulfilling precisely the gap towards the definition of a full model-assisted framework for SHM systems based on PZT sensor network design.

### 2.2. Generative Adversarial Networks for Data Synthesis

Generative adversarial neural networks derive their name from their use as generative models and their adversarial training procedure. Two separate models called the generator and the discriminator are interchangeably trained in an opposed contest during the training procedure.

In the general case, the generator is a decoder neural network, which means that it is tailored to take in a limited amount of information or features and produce a much more complex output based on that input. In the case of basic GANs, it is fed random input and expected to produce output mimicking the given data. Traditionally, these data were images. However, since the inception of GANSs, their uses have expanded beyond that. In this specific application, they will be expected to produce time-domain guided-wave-based signals.

The discriminator is a simple encoder-like classifier neural network, meaning that it takes complete signals, deconstructs them into learned features, and makes its classification decision based on these features. Its purpose is to distinguish real data samples from those created by the generator. The training goal for the generator is set as minimizing the accuracy of the discriminator. This way, interchangeably updating the weights of these two models will result in the discriminator constantly improving its capabilities to tell the real and fake data apart and give the generator progressively more information on how to better fool the discriminator. To illustrate this process, let us consider an abstraction of the training process of a GAN to create images of cats:

1. The discriminator looks for a way to tell synthetic and real images apart and ends up focusing on looking for a silhouette of the cat, as initially the generator output will mostly be random noise;
2. Knowing that the discriminator looks for silhouettes of cats, the generator adjusts to create synthetic data containing such silhouettes;
3. The discriminator now needs to find a different set of features to distinguish the samples, e.g., presence and shape of eyes;
4. The generator adjusts to suit the newly-found criteria for distinction;
5. Steps 3 and 4 keep repeating the cycle of the discriminator finding flaws in the synthetic data and the generator adjusting to fill them.

#### Style-Based GANs

One of the notable shortcomings of the original GANs is the relatively low amount of control that the user has over their output, as they are trained to generate data based on random input. StyleGAN [23,24] addresses that limitation with the introduction of “style vector”. The neural network is instead initialized with a constant vector, and the random input is fed into a trainable mapping network (MN), a multilayer perceptron that is trained to output the style vector. It is then fed into the generator at multiple points corresponding to various detail levels of the synthesized data. Since both the generator and MN are trained together, this results in the MN learning to create a mapping process between random noise and an arbitrary set of features that the generator can use as a basis for the synthetic data. These styles are applied to the model at multiple scales to influence both large-scale elements and fine-grained details, which, in the case of images, could refer to the background, color palette, or various objects, and in the case of time-domain signals, various frequency bands. Once training is done, the mapping network is no longer necessary and the values of the style vector can be manipulated directly, giving the user control over the contents of the data produced by the generator.

The descriptions of the exact neural network architectures utilized by StyleGAN, as well as the improvements made over the original version, are presented in-depth by Karras et al. [23,24].

### 2.3. GW-GAN

#### 2.3.1. Generator

**Figure 2:**
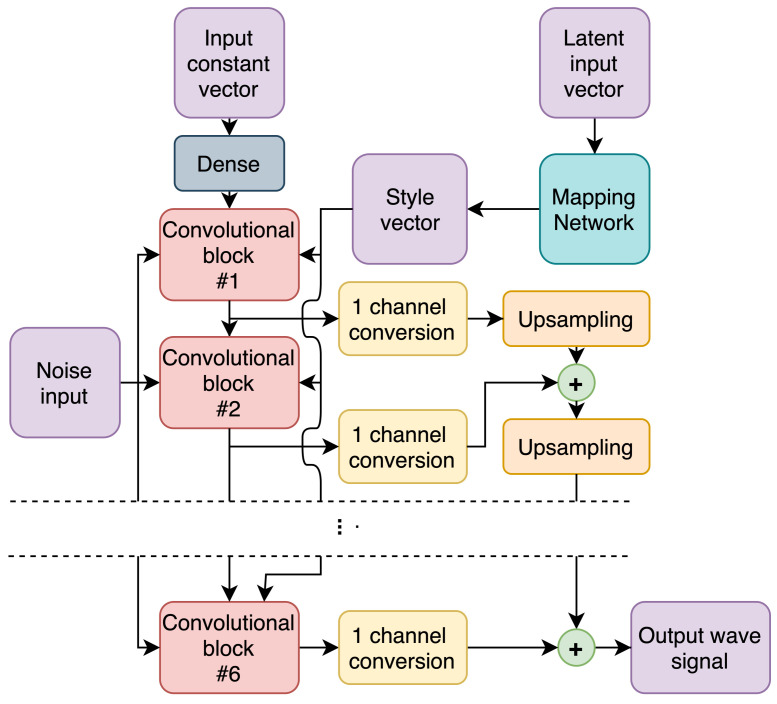
Proposed GW-GAN generator architecture.

The GW-GAN generator (presented in Figure 2) is composed of six similarly-structured convolutional blocks, containing:

1. Upsampling operation—bilinear interpolation;
2. Two one-dimensional convolutions with weight demodulation [23];
3. Two leaky rectified linear unit (ReLU) activations, one after each convolution.

The input to each of these blocks is the output of the previous one, besides the first block, which is fed a constant vector passed through a dense layer, which is a single neural layer with an arbitrary number of neurons. Additionally, each block receives supplementary inputs in the form of random noise applied to each channel, as well as a style vector corresponding to the scale of the block, as per the original styleGAN2 architecture [24].

Besides being input to the next block, the output of each block is also extracted and converted to a one-dimensional signal via a weight demodulated convolution with a single filter, followed by a tanh activation, as the desired signals can have both positive and negative values. Lastly, all these partial outputs representing multiple scales are appropriately upscaled with bilinear interpolation to match the size of the last block’s output, and summed together.

Due to the fact that output signals are oscillating and that there are various scales reflecting different frequency bands in the output signal, the number of higher-frequency components was increased by using a non-uniform upscaling approach. To this end, the upscaling operation was not used in the first block; a factor of 4 upscale was used for the next three blocks and, finally, the last two blocks upsample the signal by a factor of 2. Table 1 provides an overview of the filter counts used and the number of output samples. The regular convolution layers all have a kernel size of 3, while channel conversion layers have a kernel size of 1. The network that is used for mapping is a standard 4-hidden-layer perceptron with leaky ReLU activation functions and 128 neurons in each hidden layer.

#### 2.3.2. Discriminator

**Figure 3:**
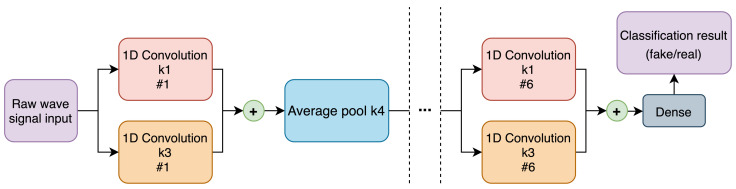
Proposed GW-GAN discriminator architecture.

Just like the generator, the discriminator is built out of six similar blocks, with its structure being the “reverse” of the generator, starting with small filter counts and ending with large ones. The other three key differences are not using weight demodulation, utilizing average-pooling operation in place of bilinear interpolation for changing the size of the signal as it passes through these individual blocks, and using skip-connections [55] instead of passing the entire residual signal across the whole network. The regular convolution layers have a kernel size of 3, while those on residual connections have a kernel size of 1. The schematic of the discriminator can be seen in Figure 3.

#### 2.3.3. Style-Finding for A Given Signal

The style vector introduced in the StyleGAN architecture allows for the direct manipulation of the output of the generator. This facilitates various synthesis options, such as adjusting individual values to change a specific characteristic, mixing and matching the styles at different scales from various signals, or performing traversals from one set of styles to another. It is also possible to find the style vector for a given image, whether to pick a specific starting point for style manipulations or to build a signal-to-style map of a labeled database, which could be then analyzed for connections between the image contents and various style vector values.

This process only utilizes the generator with its internal weights frozen throughout the procedure. To start, the generator is supplied with an arbitrary style vector, ideally one corresponding to an output similar to the desired one. Then, this vector is adjusted with a gradient descent algorithm, with its optimization function being the distance between the desired output and the actual output of the generator. Though this could be a simple mean square error between the two, it often results in the optimization getting stuck in unsatisfactory local minima. Instead, better results can be obtained by the use of perceptual distance, which is the difference between the values produced by the last convolutional layer of a robust image classifier for these two images [56].

In the case of GW-GAN, starting with random initialization turned out to be very unreliable. To address that, firstly, a base of style–signal pairs was pre-generated by the GAN by supplying it with random style vectors; then, several neural networks (with identical architecture as the discriminator, with the difference of a final dense layer with 128 outputs, matching the length of style vector) were trained on them in an attempt to create a signal–style mapping. This would allow for “guessing” the style vector for a given signal with some accuracy, resulting in somewhat close starting points for the signal-finding process. However, these attempts have not produced satisfying results. As a workaround, the starting position was selected based on this base of pre-generated style–signal pairs, by picking the style vector of the signal most closely matching the signal of interest and using mean squared error for gauging similarity.

Even with a good starting point, the signal finding process still faced several issues. First of all, as opposed to image-based object-recognition tasks, guided wave signals lack large publicly available feature extractors that could be used for perceptual difference. Secondly, the fact that the guided wave signals oscillate around y = 0 results in a problematic local minimum at this value.

Despite sub-par performance at their intended task, the previously mentioned style-guessing networks turned out to be serviceable feature extractors, and the outputs of their last convolution layers were successfully used for perceptual difference calculations. To avoid the optimization falling into the local minimum of y(x) = 0, the process is divided into two stages:

1. Stage one—with two loss components, perceptual difference, and mean squared error, strongly weighted towards the former. This way, during the first part of the backpropagation process, the network is mostly guided to produce roughly the same shape of the signal;
2. Stage two—the output is fine-tuned using only mean squared error loss to fix the remaining discrepancies between the signals.

#### 2.3.4. Training

**Figure 4:**
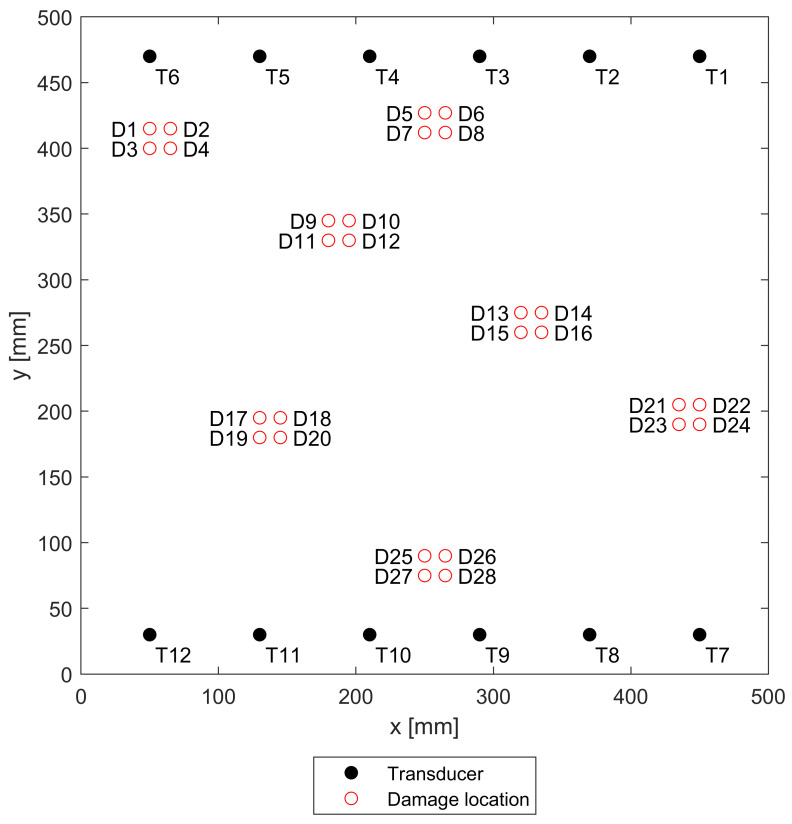
Geometry of the plate, damage, and transducer positions used in OpenGuidedWaves dataset [5].

OpenGuidedWaves dataset [5] was used in this work due to the sheer amount of data it offers, especially when it comes to the amount of diverse damage position scenarios in a controlled environment. The dataset comprises guided wave measurements performed on a square carbon-reinforced polymer plate with dimensions of 500 mm × 500 mm and 2 mm thickness. It has 12 piezoelectric transducers embedded along two of its opposite edges. Besides the wide spectrum of recorded excitation frequencies (60 kHz–240 kHz), the dataset also contains a large number of damage scenarios, with a total of 28 distinct damage states (see Figure 4), realized via attaching magnets to the monitored object. For the exact details of the plate and signal acquisition, the reader is invited to read the detailed dataset specification [5].

The original signals were generated using a Hann-filtered sine wave with five cycles as an excitation signal and were measured with 10 MHz sampling frequency, capturing a total of 13,108 samples for each signal. For the purpose of this work, only signals with 60 kHz excitation frequency were used, after several pre-processing steps necessitated by the neural network structure and a significant amount of measurement noise. The latter is problematic because the generator would also attempt to re-create the noise if presented with unfiltered data during training. Firstly, the signals were cropped to 8192 samples, and secondly, a bandpass filtration with bands at 20 and 100 kHz was performed.

Since the damage introduced to the measured object on or near the measurement path has the highest effect on the signal, the signals containing damage were handpicked. The damages in the plate were placed in groups of four, resulting in a total of seven groups for which the paths of interest had to be selected. The selection process was arbitrary, based on a perceived distance of the damage from a measurement path. The selected sensor pairs for each group were as follows:

1. T6:T10–12;
2. T3:T10–12, T4:T7–10;
3. T3:T12, T4:T10–12, T5:T7–T10, T6:T7–8;
4. T1:T10–11, T2:T9–10, T3:T7–9, T4:T7;
5. T2:T12, T3:T12, T4:T11–12, T5:T10–11, T6:T10–11;
6. T1:T7–8, T2:T7, T3:T7;
7. T1:T10, T2:T10, T3:T10, T4:T9–10, T5:T9, T6:T9.

In the above notation, T1:T10–T12 stands for sensor pairs T1:T10, T1:T11, T1:T12. Only groups 1–6 were used for training, with Group 7 being left out to validate the results of training. This resulted in a total of 160 signals used for training and 27 for validation. Each of the final models (mapping network + discriminator + generator) had around 1.8 million parameters in total and followed the same training procedures as StyleGAN2 [24]. They were trained for 350 epochs taking approximately 150 h each on an Nvidia GeForce 1050ti graphical processing unit, fitting a batch size of 8.

#### 2.3.5. Specialized Models

During development, multiple versions of the model were trained, iteratively adapting the architecture and data preparation to improve the performance gradually until arriving at the architecture and process presented in this section. For this work, three copies of the final model were trained to investigate the repeatability of the procedure. The models were trained solely on hand-picked data for damaged states. The initial plan was to have two groups of models—one trained solely on data samples containing damage and the other on baseline data. However, the models trained using only baseline data have consistently displayed sub-par performance in comparison to those trained strictly on signals containing damage, and have thus been discarded in favor of baseline signals generated using the damage-based GAN only. The hypothesized cause behind the sub-par performance of the baseline-trained GANs is the relatively low count and diversity of the baseline signals, as only 66 unique baseline scenarios were available in contrast to 160 unique path and damage combinations.

## 3. Results

### 3.1. Training Results

#### 3.1.1. Generation from Random Noise

**Figure 5:**
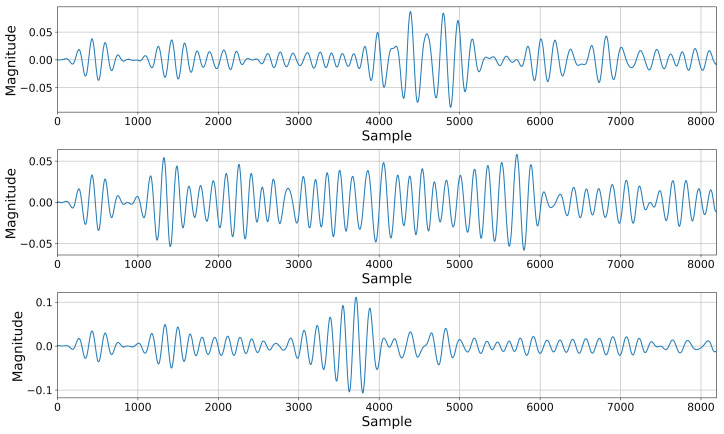
Examples of signals generated from random noise.

The simplest method of generating data with GANs is to utilize random noise as an input, exactly as is done during the training. The examples of such generation indeed resemble guided wave signals and can be seen in Figure 5. Though producing signals in this way has limited utility, it is a good way to check whether the training was successful. It is also apparent that the training avoided mode collapse—a phenomenon where the generator “collapses” into producing a single signal regardless of the input, as it is the easiest way to fool the discriminator.

#### 3.1.2. Re-Creating Validation Signals

Following the process several signals from the training data were reconstructed to verify whether the process is valid, as the generator should be able to reconstruct the signal it has seen with good accuracy.

**Figure 6:**
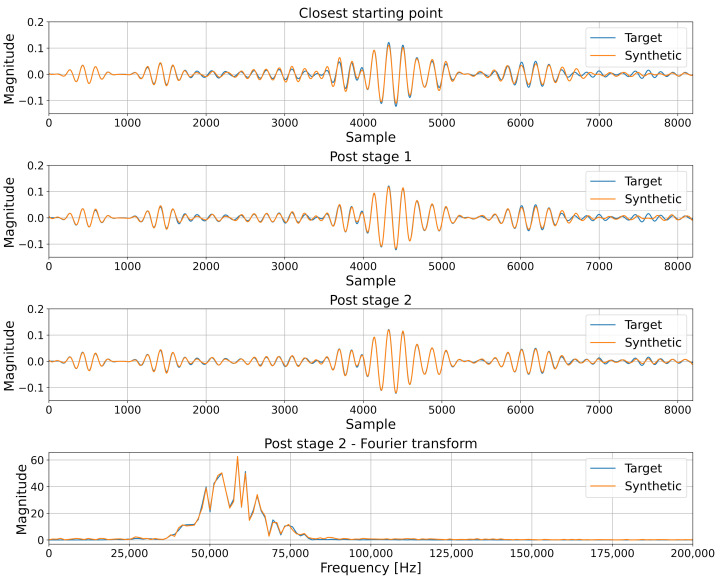
Process of re-creating a given signal from validation subset: sensor pair T6:T9 for damage position 27.

Next, the reconstruction capabilities were tested on validation signals—signals that the network has not seen during the training. The purpose of this test was to verify whether the network overfit the training dataset and would only be able to recreate the signals it has seen, or if it modeled the underlying problem and is able to generate different signals correctly. The signals were generated using the process outlined in Section 2.3.3, and an example of such reconstruction together with the two-step breakdown is presented in Figure 6.

The reconstruction accuracy is gauged by mean squared error between the original and synthetic signal, with the aggregate values presented in Table 2. Additionally, baseline comparisons with the root mean squared error as damage index have been performed for combinations of original signals and signals produced by various networks, as per Table 3.

### 3.2. Classification Based on Synthetic Data

#### 3.2.1. Setup and Data Synthesis Strategy

As the main purpose of this signal synthesis research is to facilitate the creation of extra data for classifier training, a simple testing scenario was set up to validate the usability of such synthetic signals. The classifiers were trained solely on synthetic data and then validated on real signals to check whether systems trained in this manner can still perform adequately when faced with real data.

The training signals were generated by the network with the best reconstruction performance, based on damage positions 5–8 (see Figure 4) for sensor pairs T3:T10–12 and T4:T7–T10. The style vectors needed for the exact reconstruction of baseline signals and signals containing damage on these paths have been mixed with random ratios to increase the variation between the signals and create signals differing from the original ones on these paths. In effect, this results in creating “almost undamaged paths” where the ratio favors baseline signals and “lightly damaged paths” where the ratio favors signals containing damage. These ratios were drawn from a uniform random distribution, ranging from 0.15:0.85 to 0.3:0.7 for both scenarios. When it comes to the features used for classification, a set of the following four reference-based damage indices was chosen:

- $DI_{XCO}$: Pearson cross-correlation estimate obtained between baseline and the signal for the lag value equal to 0 [57];
- $DI_{RMS}$: Root mean square value of the difference between the signal and the baseline;
- $DI_{IP}$: Instantaneous-phase-based temperature compensation damage index [58].;
- $DI_{ENV}$: Normalized squared error between envelopes of the signal and the baseline. Envelopes are calculated using Hilbert transform [57];
- $DI_{MXC}$: Maximum value of Pearson cross-correlation estimate obtained for all possible lags between signal and the baseline [57].

All the reference signals were generated as “almost undamaged paths”. If the main signal was from that group as well, it was labeled as undamaged; if it was from the “lightly damaged paths” group, it was labeled as damaged. Thus, for each of the 28 damage position and sensor path combinations, a total of 20 signal pairs were produced: one half representing a damaged state and the other half representing the intact state.

#### 3.2.2. Results

The classification was initially performed by random forest classifier (100 estimators, Gini criterion, no max depth), which was chosen due to its simplicity and good performance in feature-based classification problems. To broaden the testing, the procedure was also done for a simple multi-layer perceptron classifier (two hidden layers with 100 neurons each, ReLU activation, ADAM optimizer with 1 × 10^−3^ initial learning rate). The configurations for these two classifiers were picked arbitrarily without going into fine-tuning and classification method optimization, as the goal of this experiment was to check whether a classifier trained on synthetic data can later perform well on real data, not exactly how well a classifier can perform on this specific batch of synthetic and real data.

To consider the repeatability of the results, each of the classifiers was trained 100 times. Without exceptions, all classifiers achieved 100% accuracy on the validation set due to the large margin of separation between the classes in feature space, making it a trivial classification problem. It is, however, worth noting that using this synthetic data, it is certainly possible to train a classifier that will not have good accuracy if the decision boundaries found in the training process are placed too close to one of the classes in the feature space, making the problem prone to overfitting.

## 4. Discussion

**Figure 7:**
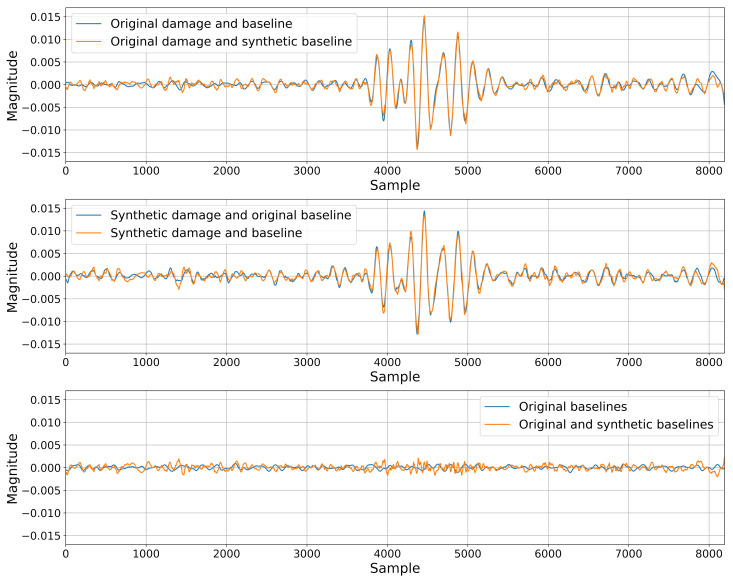
Residuals for synthetic and original signal pairs from validation subset: sensor pair T1:T10 for damage position 25.

The guided wave signal synthesis appears to perform well at every step, producing believable signals via random generation (Figure 5) and accurately reconstructing given signals that it had seen during training (Figure 6) as well as ones that it had not yet encountered (Figure 7), carrying relevant information about present damage or lack thereof. The fact that it was able to accurately reconstruct the damage scenarios that it had not previously seen suggests that any given damage scenario may exist in the space modeled by the GAN. Currently generating signals for arbitrarily picked damage positions has not been tested; however, formulating a robust control method would allow for a synthesis of other damage scenarios or even sensor paths placed in any position on the monitored object.

As seen in Section 3.2, these synthetic signals can be used to train classifiers that will later work on real data; however, it is important to keep in mind that the classification test presented in this paper is a limited proof-of-concept. The feasibility of this approach should be evaluated and analyzed more thoroughly with large-scale tests before any industrial applications. Additionally, only feature-based classification was tested in this work. Though it is the usual approach for SHM using guided waves, it will be interesting to see the effects that this synthetic data might have on methods that work with raw signals instead, e.g., deep neural networks.

Though all three trained networks work to some extent—showing that it is a repeatable and reliable process—there is significant variability in how well exactly they perform. These differences can be noted from Table 2. The cause behind this is the inherently random nature of the training process. Though ideally, these performance gaps would be lower, they are not necessarily a significant issue. The training procedure for this network (briefly outlined in Section 2.2 is open-ended. There is no easily-defined point where the training ends because the model will not become any better, and the training length, in this case, was picked arbitrarily, mostly due to time constraints. Due to this, the models with worse performance can generally be improved by simply prolonging their training from the current point.

Due to the non-recursive nature of the generator (all the individual samples are generated independently of each other), the signal synthesis step is extremely fast; obtaining signals represented by given 100 style vectors is a matter of 177 milliseconds on a nVidia GeForce 1050Ti. Unfortunately, in the current implementation, the other steps are not quite as fast, especially finding the exact style vector that is needed to reconstruct a given signal, which takes around 18 minutes per signal on the same GPU. Finding ways to speed up this process will be an essential part of future research concerning this topic, as the usefulness of this method hinges not only on the quality of the results it produces, but also on the time required to obtain these results.

Besides generating new signals, the GW-GAN was shown to be able to reconstruct the samples from its training dataset with high accuracy. The implication of that is that if this accuracy is satisfying, the network could be used as a compression tool. In these specific scenarios, signals with 8192 samples are represented by style vectors with just 128 values, resulting in 98.44% compression. That being said, as previously mentioned, the style-finding process is currently slow and would likely need to be considerably sped up to be considered a valid compression method.

## 5. Conclusions

Three separate instances of GW-GAN were successfully trained. They were able to reconstruct both signals drawn from their training dataset and previously unseen signals, showing that with properly formulated control, these networks will be able to generate signals with arbitrary sensor and damage placement. The generated signals are of diagnostic quality, as they correctly carry damage index information. Proof-of-concept classification using models trained solely on synthetic data resulted in correct classification of real signals on different measurement paths. Having to describe the signals with the style vector also makes the model a potent compression tool, with a roughly 98.44% compression rate.

## Figures and Tables

**Table 1:** Filter count, output samples, and approximate frequency limit as a fraction of the sampling frequency for convolutional blocks in the generator.

Block ID	1	2	3	4	5	6
Filter count	384	192	144	96	48	24
Output samples	32	128	512	2048	4096	8192
Max frequency	1.95 × 10${}^{-3}$	7.8 × 10${}^{-3}$	3.1 × 10${}^{-2}$	1.25 × 10${}^{-1}$	2.5 × 10${}^{-1}$	5 × 10${}^{-1}$

**Table 2:** Reconstruction mean square error across damaged validation signals.

GAN #	Mean	Std	Min	Max
1	6.33 × 10${}^{-6}$	4.46 × 10${}^{-6}$	1.21 × 10${}^{-6}$	1.78 × 10${}^{-5}$
2	1.92 × 10${}^{-6}$	3.12 × 10${}^{-6}$	3.06 × 10${}^{-7}$	1.34 × 10${}^{-5}$
3	8.49 × 10${}^{-7}$	9.45 × 10${}^{-7}$	2.23 × 10${}^{-7}$	4.71 × 10${}^{-6}$

**Table 3:** RMSE damage index values for original and synthetic signals for validation paths.

Signal 1	Signal 2	Mean RMSE
Original baseline series 1	Original baseline series 2	4.29 × 10${}^{-4}$
Original damaged	Original baselines series 1	2.27 × 10${}^{-3}$
Synthetic damaged net 1	Original baselines series 1	3.18 × 10${}^{-3}$
Synthetic damaged net 2	Original baselines series 1	2.55 × 10${}^{-3}$
Synthetic damaged net 3	Original baselines series 1	2.21 × 10${}^{-3}$

## Data Availability

The OpenGuidedWaves dataset that was used for training of the models can be found under http://openguidedwaves.de/downloads/ accessed on 9 December 2019. Guided wave basic measurement data was used.
